# Quantum metrology with unitary parametrization processes

**DOI:** 10.1038/srep08565

**Published:** 2015-02-24

**Authors:** Jing Liu, Xiao-Xing Jing, Xiaoguang Wang

**Affiliations:** 1 Zhejiang Institute of Modern Physics, Department of Physics, Zhejiang University, Hangzhou 310027, China; 2 Synergetic Innovation Center of Quantum Information and Quantum Physics, University of Science and Technology of China, Hefei, Anhui 230026, China

## Abstract

Quantum Fisher information is a central quantity in quantum metrology. We discuss an alternative representation of quantum Fisher information for unitary parametrization processes. In this representation, all information of parametrization transformation, i.e., the entire dynamical information, is totally involved in a Hermitian operator 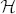. Utilizing this representation, quantum Fisher information is only determined by 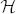 and the initial state. Furthermore, 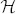 can be expressed in an expanded form. The highlights of this form is that it can bring great convenience during the calculation for the Hamiltonians owning recursive commutations with their partial derivative. We apply this representation in a collective spin system and show the specific expression of 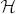. For a simple case, a spin-half system, the quantum Fisher information is given and the optimal states to access maximum quantum Fisher information are found. Moreover, for an exponential form initial state, an analytical expression of quantum Fisher information by 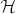 operator is provided. The multiparameter quantum metrology is also considered and discussed utilizing this representation.

How to precisely measure the values of physical quantities, such as the phases of light in interferometers, magnetic strength, gravity and so on, is always an important topic in physics. Obtaining high-precision values of these quantities will not only bring an obvious advantage in applied sciences, including the atomic clocks, physical geography, civil navigation and even military industry, but also accelerate the development of fundamental theories. One vivid example is the search for gravitational waves. Quantum metrology is such a field attempting to find optimal methods to offer highest precision of a parameter that under estimation. In recently decades, many protocols and strategies have been proposed and realized to improve the precisions of various parameters^1^,^2^,^3^,^4^,^5^,^6^,^7^,^8^,^9^,^10^,^11^,^12^,^13^,^14^,^15^,^16^,^17^,^18^,^19^,^20^,^21^,^22^. Some of them can even approach to the Heisenberg limit, a limit given by the quantum mechanics, showing the power of quantum metrology.

Quantum Fisher information is important in quantum metrology because it depicts the theoretical lowest bound of the parameter's variance according to Cramér-Rao inequality^23^,^24^. The quantum Fisher information for parameter *α* is defined as *F* = Tr(*ρL*^2^), where *ρ* is a density matrix dependent on *α* and *L* is the symmetric logarithmic derivative (SLD) operator and determined by the equation *∂_α_ρ* = (*ρL* + *Lρ*)/2. For a multiparameter system, the counterpart of quantum Fisher information is called quantum Fisher information matrix 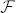, of which the element is defined as 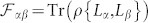, where *L_α_*, *L_β_* are the SLD operators for parameters *α* and *β*, respectively.

Recently, it has been found^27^ that quantum Fisher information can be expressed in an alternative representation, that all information of parametrization process in quantum Fisher information is involved in a Hermitian operator 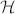. This operator characterizes the dynamical property of the parametrization process, and totally independent of the selection of initial states. Utilizing this representation, the quantum Fisher information is only determined by 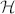 and the initial state.

In this report, we give a general expression of quantum Fisher information and quantum Fisher information matrix utilizing 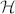 operator. For a unitary parametrization process, 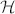 can be expressed in an expanded form. This form is particularly useful when the Hamiltonian owns a recursive commutation relation with its derivative on parameter estimation. We calculate the specific expression of 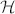 in a collective spin system, and provide an analytical expression of quantum Fisher information in a spin-half system for any initial state. Based on this expression, all optimal states to access maximum quantum Fisher information are found in this system. Furthermore, considering this spin-half system as a multiparameter system, the quantum Fisher information matrix, can be easily obtained by the known form of 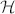 in single parameter estimations. On the other hand, inspired by a recent work^28^, for an exponential form initial state, we provide an analytical expression of quantum Fisher information using 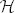 operator. A demonstration with a spin thermal initial state is given in this scenario. The maximum quantum Fisher information and the optimal condition are also discussed.

## Results

### Quantum Fisher information with 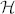 operator

For a general unitary parametrization transformation, the parametrized state *ρ*(*α*) is expressed by *ρ*(*α*) = *U*(*α*)*ρ*_0_*U*^†^(*α*), where *ρ*_0_ is a state independent of *α*. In this paper, since the parameter *α* is only brought by *U*(*α*), not the initial state *ρ*_0_, we use *U* instead of *U*(*α*) for short. Denote the spectral decomposition of *ρ*_0_ as 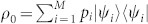, where *p_i_* and |*ψ_i_*〉 are the *i*th eigenvalue and eigenstate of *ρ*_0_ and *M* is the dimension of the support of *ρ*_0_. It is easy to see that *p_i_* and *U*|*ψ_i_*〉 are the corresponding eigenvalue and eigenstate of *ρ*(*α*), respectively. The quantum Fisher information for *ρ*(*α*) can then be expressed by^29^,^30^

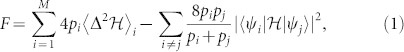
where^25^,^26^

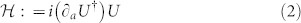
is a Hermitian operator since the equality (*∂_α_U*^†^)*U* = −*U*^†^(*∂_α_U*). Meanwhile, 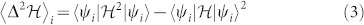
is the variance of 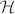 on the *i*th eigenstate of *ρ*_0_. When *∂_α_U* commutes with *U*, 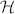 can be explained as the generator of the parametrization transformation^27^. The expression (1) of quantum Fisher information is not just a formalized representation. The operator 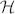 is only determined by the parametrization process, that is the dynamics of the system or the device. For a known dynamical process of a parameter, i.e., known system's Hamiltonian, 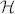 is a settled operator and can be obtained in principle. In this representation, the calculation of quantum Fisher information is separated into two parts: the diagonalization of initial state and calculation of 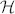. For a general 2-dimensional state, the quantum Fisher information reduces to 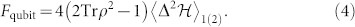
The subscript of the variance can be chosen as 1 or 2 as any Hermitian operator's variances on two orthonormal states are equivalent in 2-dimentional Hilbert space. For a pure state, the quantum Fisher information can be easily obtain from Eq. (4) with taking the purity Tr*ρ*^2^ = 1 and the variance on that pure state, i.e., ref. 27

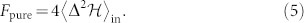
Namely, the quantum Fisher information is proportional to the variance of 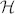 on the initial state. In this scenario, denote the initial state *ρ*_0_ = |*ψ*_0_〉〈*ψ*_0_|, the quantum Fisher information can be rewritten into 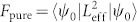, with the effective SLD operator 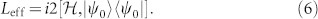
For a well applied form of parametrization transformation *U* = exp(−*itH_α_*)^27^, where 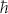 has been set as 1 in Planck unit, and being aware of the equation 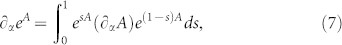

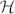
 can then be expressed by 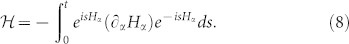
Defining a superoperator *A*^×^ as *A*^×^(·) : = [*A*, ·], 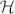 can be written in an expanded form 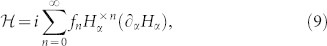
where the coefficient 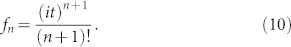
In many real problems, the recursive commutations in Eq. (9) can either repeat or terminate^28^, indicating an analytical expression of 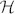. Thus, this representation of quantum Fisher information would be very useful in these problems. For the simplest case that *H_α_* = *αH*, all terms vanish but the first one, then 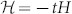. When [*H_α_*, *∂_α_H_α_*] = *C*, with *C* a constant matrix or proportional to *H_α_*, only the first and second terms remain. In this case, 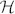 reduces to −*t*(∂*_α_H_α_* + *itC*/2). A more interesting case is that [*H_α_*, ∂*_α_H_α_*] = *c*∂*_α_H_α_*, with *c* a nonzero constant number, then 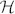 can be written in the form 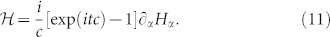
 In the following we give an example to exhibit Eq. (9). Consider the interaction Hamiltonian of a collective spin system in a magnetic field 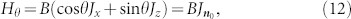
where 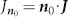 with ***n***_0_ = (cos *θ*, 0, sin *θ*)^T^ and ***J*** = (*J_x_*, *J_y_*, *J_z_*)^T^. *B* is the amplitude of the external magnetic field and *θ* is the angle between the field and the collective spin. Here 
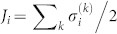
 for *i* = *x*, *y*, *z* with 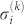 the Pauli matrix for *k*th spin. Taking *θ* as the parameter under estimation, 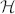 can be expressed by 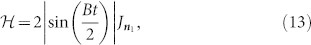
where 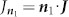 with the vector 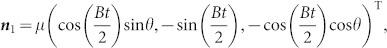
where *µ* = sgn(sin(*Bt*/2)) is the sign function and ***n***_1_ is normalized.

The operator 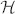 for Hamiltonian (12) may be also available to be solved using the procedure in Ref. 27, in the (2j + 1)-dimensional eigenspace of *H_θ_* (j is the total spin). In principle, the eigenstates of *H_θ_* can be found by rotating the Dicke state into the same direction of *H_θ_*. However, even one can analytically obtain all the eigenvalues and eigenvectors, it still requires a large amount of calculations to obtain 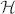, especially when the spin numbers are tremendous. Comparably, utilizing Eq. (9), it only takes a few steps of calculation, which can be found in the method. This is a major advantage of the expanded form of 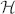.

**Figure 1:**
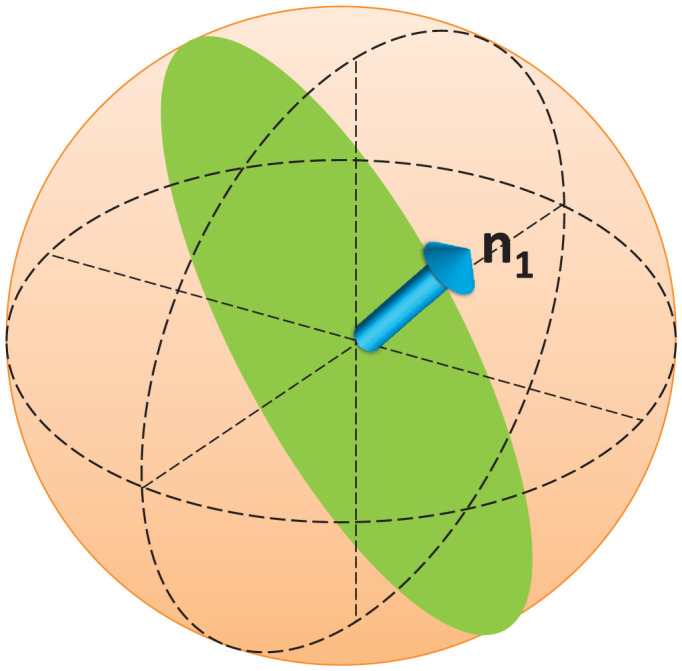
Optimal states to access maximum quantum Fisher information in a spin-half system. The blue arrow represents the vector ***n***_1_ and all vectors in the green plane are vertical to ***n***_1_. All the states in the joint ring of green plane and Bloch sphere's surface can access maximum quantum Fisher information.

Utilizing Eq. (13), one can immediately obtain the form of 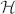 for a spin-half system 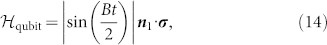
with ***σ*** = (*σ_x_*, *σ_y_*, *σ_z_*)^T^, which was also discussed in the Hamiltonian eigenbasis in Ref. 27. For any 2-dimensional state, based on Eq. (4), the quantum Fisher information can be expressed by 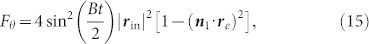
where ***r***_in_ = (〈*σ_x_*〉, 〈*σ_y_*〉, 〈*σ_z_*〉)^T^ is the Bloch vector of the initial state *ρ*_0_ and ***r***_e_ is the Bloch vector of any eigenstate of *ρ*_0_. For pure states, there is ***r***_e_ = ***r***_in_ and |***r***_in_| = 1. Since the Bloch vector of a 2-dimensional state satisfies |***r***_in_| ≤ 1, it can be found that the maximum value of Eq. (15) is 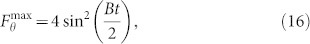
which can be saturated when |***r***_in_| = 1 and ***n***_1_ · ***r***_in_ = 0, namely, the optimal state to access maximum quantum Fisher information here is a pure state perpendicular to ***n***_1_, as shown in Fig. 1. In this figure, the yellow sphere represents the Bloch sphere and the blue arrow represents the vector ***n***_1_. It can be found that all states on the joint ring of the green plane and surface of Bloch sphere can access the maximum quantum Fisher information, i.e., all states on this ring are optimal states. One simple example is ***r***_opt_ = ***n***_0_, and another one is the superposition state of two eigenstates of 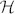
22,27.

Alternatively, *B* could be the parameter that under estimation. In the spin-half case, with respect to *B*, 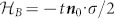, then the quantum Fisher information can be expressed by 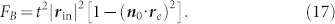
The optimal states to access the maximum value 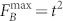 are the pure states vertical to ***n***_0_.

### Exponential form initial state

For an exponential form initial state *ρ*_0_ = exp(*G*_0_), the parametrized state reads 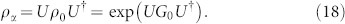
Recently, Jiang^28^ studied the quantum Fisher information for exponential states and gave a general form of SLD operator. In his theory, the SLD operator can be expanded as 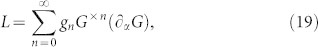
where the coefficient 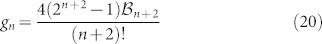
for even *n* and *g_n_* vanishes for odd *n*. Here 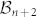 is the (*n* + 2)th Bernoulli number and in our case, *G* = *UG*_0_*U*^†^. Through some straightforward calculation, the derivative of *G* on *α* reads 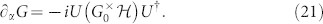
Based on this equation, the *n*th order term in Eq. (19) is 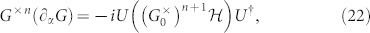
where 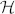 is given by Eq. (9). Generally, it is known that the quantum Fisher information reads 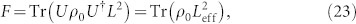
where the effective SLD operator *L*_eff_ = *U*^†^*LU*. The effective SLD operator for pure states is already shown in Eq. (6). Substituting Eq. (22) into Eq. (19), the effective SLD operator can be expanded as 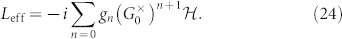
In most mixed states cases, to obtain quantum Fisher information, the diagonalization of initial state is inevitable, which is the reason why the usual form of quantum Fisher information is expressed in the eigenbasis of density matrix. Thus, it is worth to study the expression of effective SLD operator and quantum Fisher information in the eigenbasis of *G*_0_. We denote the *i*th eigenvalue and eigenstate of *G*_0_ as *a_i_* and |*ϕ_i_*〉, and in the eigenbasis of *G*_0_, the element of 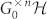 satisfies the recursion relation 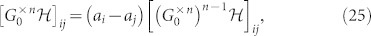
where [·]*_ij_* : = 〈*ϕ_i_*| · |*ϕ_j_*〉. Utilizing this recursive equation, a general formula of *n*th order term can be obtained, 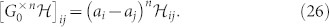
Substituting above equation into the expression of *L*_eff_ and being aware of the equality 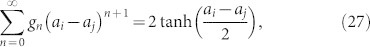
the element of effective SLD operator in Eq. (24) can be written as 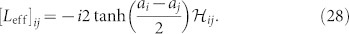
Based on the equation 
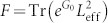
, the quantum Fisher information in the eigenbasis of *G*_0_ can finally be expressed by 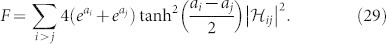
This is one of the main results in this paper. In some real problems, the eigenspace of *G*_0_ could be find easily. For instance, the eigenspace of a bosonic thermal state is the Fock space. Thus, as long as the formula of 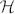 in Fock space is established, the quantum Fisher information can be obtained from Eq. (29).

**Figure 2:**
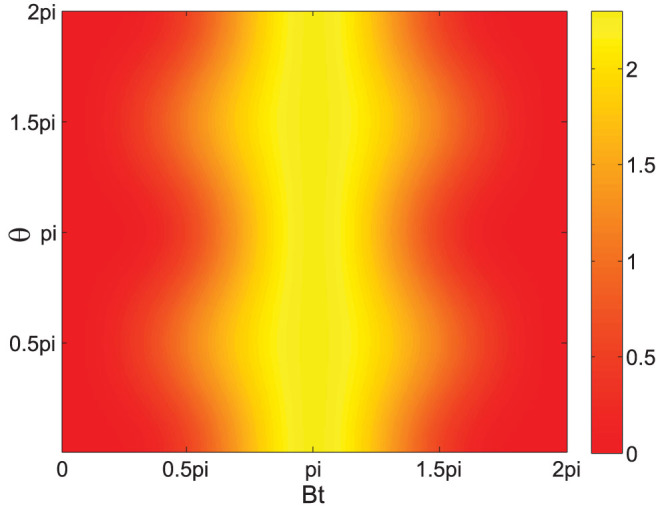
Quantum Fisher information as a function of *Bt* and *θ*. The initial state is a spin-half thermal state and the temperature is set as *T* = 1 here.

Now we exhibit Eq. (29) with a spin-half thermal state. The initial state is taken as 
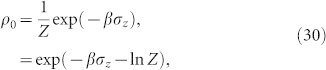
where *β* = 1/(*k*_b_*T*) with *k*_b_ the Boltzmann constant and *T* the temperature. In Planck unit, *k*_b_ = 1. The partition function reads *Z* = Tr[exp(−*βσ_z_*)] = 2 cosh *β*. In this case, *G*_0_ = −*βσ_z_* − ln *z*. Denoting the eigenstates of *σ_z_* as |0〉 and |1〉, i.e., *σ_z_* = |0〉〈0| − |1〉〈1|, the eigenvalues of *G*_0_ read *a*_1_ = −*βσ_z_* − ln *z* and *a*_2_ = *βσ_z_* − ln*z*. The parametrization process is still taken as *H_θ_* = *B****n***_0_ · ***σ***/2 with *θ* the parameter under estimation, indicating that 
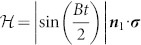
, then the squared norm of the off-diagonal element of 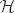 in the eigenbasis of *σ_z_* reads 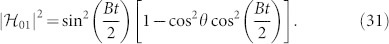
Immediately, the quantum Fisher information can be obtained from Eq. (29) as 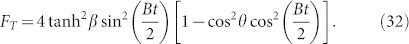
The maximum value of above expression is obtained at *Bt* = (4*k* + 1)*π* for *k* = 0, 1, … and 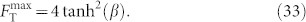
 From this equation, one can see that the value of maximum quantum Fisher information is only affected by the temperature. With the increase of temperature, the maximum value reduces. In the other hand, quantum Fisher information in Eq. (32) is related to *Bt* and *θ*. Fig. 2 shows the quantum Fisher information as a function of *Bt* and *θ*. The values of *Bt* and *θ* are both within [0, 2*π*] in the plot. The temperature is set as *T* = 1 here. From this figure, it can be found that the maximum quantum Fisher information is robust for *θ* since it is always obtained at *Bt* = *π* for any value of *θ*. Furthermore, this optimal condition of *Bt* is independent of temperature. With respect to *Bt*, there is a large regime near *Bt* = *π* in which the quantum Fisher information's value can surpass 2, indicating that the quantum Fisher information can be still very robust and near its maximum value even when *Bt* is hard to set exactly at *π*.

### Multiparameter processes

For a multiparameter system, the element of quantum Fisher information matrix in Ref. 30 can also be written with 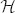 operator, 
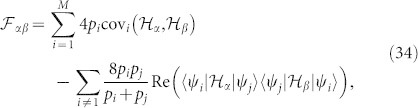
where *U* is dependent on a series of parameters *α*, *β* and so on, and 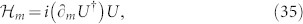
with the index *m* = *α*, *β*, …. The covariance matrix on the *i*th eigenstate of initial state is defined as 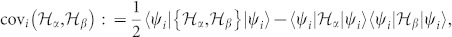
with {·, ·} the anti-commutation. For a single qubit system, Eq. (34) reduces to 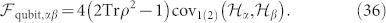
Similarly with the single-parameter scenario, the subscript in Eq. (36) can be chosen as 1 or 2 since the covariance for two Hermitian operators are the same on two orthonormal states in 2-dimensional Hilbert space. From this equation, the element of quantum Fisher information matrix for pure states can be immediately obtained as 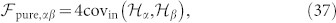
namely, for pure states, the element of quantum Fisher information matrix is actually the covariance between two 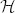 operators on the initial state. When the total Hamiltonian can be written as 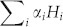 and [*H_i_*, *H_j_*] = 0 for any *i*, *j*, above equation can reduce to the covariance between *H_i_* and *H_j_*^31^. For the diagonal elements, they are exactly the quantum Fisher information for the corresponding parameters.

For multiparamter estimations, the Cramér-Rao bound cannot always be achieved. In the scenario of pure states, the condition of this bound to be tight is Im〈*ψ*_out_|*L_α_L_β_*|*ψ*_out_〉 = 0, ∀*α*, *β*^32^,^33^. Here |*ψ*_out_〉 is dependent on the parameter under estimation. In the unitary parametrization, |*ψ*_out_〉 = *U*|*ψ*_0_〉 and this condition can be rewritten into 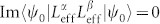, ∀*α*, *β*. Here 
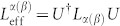
 is the effective SLD operator for parameter *α*(*β*). Utilizing Eq. (6), this condition can be expressed in the form of 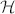 operator, 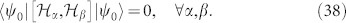
In other word, 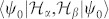 needs to be a real number for any *α* and *β*. When 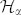 commutes with 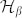 for any *α* and *β*, above condition can always be satisfied for any initial state.

Generally, for the unitary parametrization process, the element of quantum Fisher information matrix can be expressed by 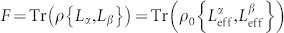. From the definition equation of SLD, one can see that 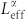 satisfies the equation ∂*_θ_ρ* = *U*{*ρ*_0_, *L*_eff_}*U*^†^/2. The quantum Fisher information matrix has more than one definitions. One alternative candidate is using the so-called Right Logarithmic Derivative (RLD)^24^,^34^,^35^, which is defined as ∂*_α_ρ* = *ρR_α_*, with *R_α_* the RLD. The element of RLD quantum Fisher information matrix can be written as 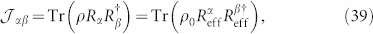
where the effective RLD reads 
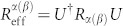
. For a unitary parametrization process, assuming the initial state has nonzero determinant, 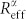 can be expressed by 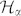 and the initial state *ρ*_0_, i.e., 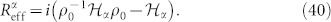
With this equation, the element of RLD quantum Fisher information matrix can be expressed by 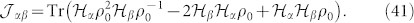
When the parametrization process is displacement, this equation can reduces to the corresponding form in Ref. 35. For pure states, the element reads 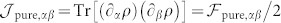. Recently, Genoni *et al.*^35^ proposed a most informative Cramér-Rao bound for the total variance of all parameters under estimation. From the relation between 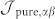 and 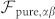, one can see that 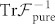 is always larger than 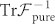, namely, the SLD Cramér-Rao bound is always more informative than the RLD counterpart in this scenario.

We still consider the spin-half system with the Hamiltonian *H* = *B****n***_0_ · ***σ***/2. Take both *B* and *θ* as the parameters under estimations. First, based on aforementioned calculation, the 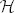 operator for *B* and *θ* read 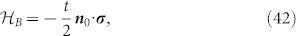

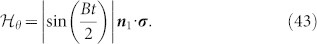


Based on the property of Pauli matrices {***n***_0_ · ***σ***, ***n***_1_ · ***σ***} = 2***n***_0_ · ***n***_1_, the anti-commutation in the covariance reads 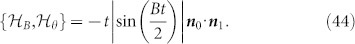
For a pure initial state, the off-diagonal element of the quantum Fisher information matrix is expressed by 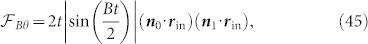
where ***r***_in_ is the Bloch vector of the initial pure state and the equality ***n***_0_ · ***n***_1_ = 0 has been used. When the initial pure state is vertical to ***n***_0_ or ***n***_1_, this off-diagonal element vanishes. Compared with the optimal condition for maximum quantum Fisher information for *B* and *θ* individually, the Bloch vector ***n***_2_ = ***n***_0_ × ***n***_1_ can optimize both the diagonal elements of quantum Fisher information matrix and vanish the off-diagonal elements. However, all above is only necessary conditions for the achievement of Cramér-Rao bound. To find out if the bound can be really achieved, the condition (38) needs to be checked. In this case, 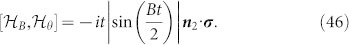
With this equation, condition (38) reduces to ***n***_2_ · ***r***_in_ = 0, i.e., to make the Cramér-Rao bound achievable, the Bloch vector of the initial state needs to in the plane of ***n***_0_ and ***n***_1_. Unfortunately, ***n***_2_ is not in this plane. Thus, *B* and *θ* cannot be optimally joint measured simultaneously.

In the plane constructed by ***n***_0_ and ***n***_1_, any Bloch vector of pure state can be written as ***r***_in_ = ***n***_0_ cos *ϕ* + ***n***_1_ sin *ϕ*, then we have 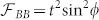, 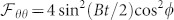, and 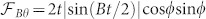. From these expressions, one can see that the determinant of quantum Fisher information matrix is zero, i.e., det 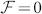. This fact indicates that, utilizing any pure state in this plane, the variances of *B* and *θ* cannot be estimated simultaneously through the Cramér-Rao theory.

## Discussion

We have discussed the quantum Fisher information with unitary parametrization utilizing an alternative representation. The total information of the parametrization process is involved in a 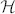 operator in this representation. This operator is totally determined by the parameter and parametrization transformation *U*. As long as the parameter and transformation are taken, 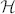 is a settled operator and independent of the initial state. More interestingly, 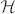 can be expressed in an expanded form. For the Hamiltonians owning recursive commutations with their partial derivative on the parameter under estimation, this expanded form shows a huge advantage. Utilizing this representation, we give a general analytical expression of quantum Fisher information for an exponential form initial state. Moreover, we have also studied the 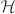 representation in multiparameter processes. The condition of Cramér-Rao bound to be achievable for pure states are also presented in the form of 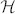 operator. In addition, we give the 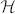 representation of Right Logarithmic Derivative and the corresponding quantum Fisher information matrix.

As a demonstration, we apply this representation in a collective spin system and show the expression of 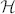. Furthermore, we provide an analytical expression of quantum Fisher information in a spin-half system. If we consider this system as a multiparameter system, the corresponding quantum Fisher information matrix can also be straight-forwardly obtained by this representation. From these expressions, one can find the optimal states to access the maximum quantum Fisher information. For the parameter *B*, the optimal state is a pure state vertical to ***n***_0_, and for the parameter *θ*, the optimal one is also a pure state, but vertical to ***n***_1_. By analyzing the off-diagonal element of quantum Fisher information matrix, the states to optimize the diagonal elements and make the off-diagonal elements vanish are found. However, these states fail to satisfy the condition of achievement. Thus, *B* and *θ* cannot be optimally jointed measured.

## Methods

### Collective spin system in a magnetic field

For the Hamiltonian (12), its derivative on parameter *θ* is 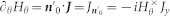 with the vector 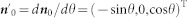. Based on Eq. (9), 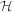 can be written as 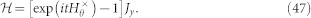
It is worth to notice that 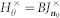, then 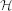 is 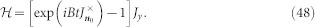
Being aware of the commutation relations 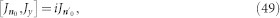

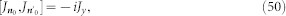
one can straightforwardly obtain the *n*th order term as below 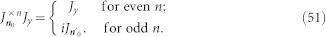
With this equation, 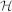 can be expressed by 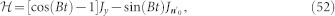
equivalently, it can be written in a inner product form: 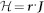, where the elements of ***r*** read *r_x_* = sin(*Bt*) sin *θ*, *r_y_* = cos(*Bt*) − 1 and *r_z_* = − sin(*Bt*) cos *θ*. After the normalization process, 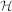 is rewritten into the form of Eq. (13).

For a spin-half system, the quantum Fisher information can be expressed by 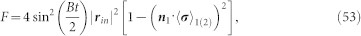
where ***r***_in_ is the Bloch vector of *ρ*_0_ and can be obtained through the equation 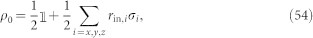
with 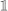 the identity matrix. 〈***σ***〉*_i_* = (〈*σ_x_*〉*_i_*, 〈*σ_y_*〉*_i_*, 〈*σ_z_*〉*_i_*)^T^ is the vector of expected values on the *i*th (*i* = 1, 2) eigenstate of *ρ*_0_. It can also be treated as the Bloch vector of the eigenstates. In previous sections, we denote ***r***_e_ : = 〈***σ***〉*_i_*.

## Author Contributions

X.W. and J.L. contributed the idea. J.L. performed the calculations and prepared the figures. X.J. checked the calculations. J.L. wrote the main manuscript and X.W. made an improvement. All authors contributed to discussion and reviewed the manuscript.
